# Transplantation of Refrozen Ovarian Cortical Strips Retrieved from a Cryopreserved Whole Ovary: Proof of Feasibility

**DOI:** 10.3390/jcm11174942

**Published:** 2022-08-23

**Authors:** Camille Hossay, Céline Pirard, Pascale Laurent, Candice Kluyskens, Jacques Donnez, Marie-Madeleine Dolmans

**Affiliations:** 1 Pôle de Recherche en Gynécologie, Institut de Recherche Expérimentale et Clinique, Université Catholique de Louvain, 1200 Brussels, Belgium; 2 Gynecology Department, Cliniques Universitaires Saint-Luc, 1200 Brussels, Belgium; 3 Society for Research into Infertility, 1150 Brussels, Belgium

**Keywords:** whole ovary cryopreservation, refreezing, ovarian tissue transplantation, pelvic radiotherapy, ovarian transposition

## Abstract

We report successful clinical outcomes after transplantation of refrozen-rethawed cortical strips from a cryopreserved whole ovary in a patient diagnosed with stage IIIb rectal adenocarcinoma. Whole ovary cryopreservation was proposed as a fertility preservation strategy in 2006 prior to radiotherapy, chemotherapy and oncological surgery. To allow for minimal residual disease screening before ovarian reimplantation, the whole ovary was thawed and dissected into cortical strips. While awaiting the results, the majority of the cortical strips were refrozen. These refrozen-rethawed cortical strips were laparoscopically grafted to 2 sites: the previously irradiated pelvic cavity and the non-irradiated extrapelvic cavity. Ovarian function resumption was assessed by recovery of menses, hormone levels, ultrasound and oocyte pick-up following controlled ovarian stimulation (COS). Restoration of ovarian function occurred 6 months after reimplantation, with recovery of menses and estradiol secretion. A total of 12 cycles were followed by the IVF department. A second reimplantation was performed 1.5 years later, since the grafts were found to have stopped functioning for >3 consecutive months. Overall, 3 fertilizable oocytes were retrieved transabdominally from the extrapelvic graft following COS, yielding 2 embryos and culminating in one fresh embryo transfer, but no pregnancy. Concerning the reimplantation site, no ovarian activity was detected in the graft placed in the previously irradiated pelvic cavity. Indeed, only fibrotic-looking tissue was observed in the pelvic site at second laparoscopy 1.5 years later, while ovarian activity was noted in the extrapelvic graft, showing a large antral follicle. All in all, transplantation of refrozen-rethawed cortical strips from a cryopreserved whole ovary can lead to ovarian function resumption and embryo development if grafted to a non-irradiated field.

## 1. Introduction

Fertility preservation programs are now an integral part of the medical care of cancer patients. In prepubertal girls or when life-saving therapy cannot be postponed, ovarian tissue cryopreservation is the only available option to preserve fertility [1,2,3]. Ovarian endocrine resumption after transplantation of frozen-thawed ovarian tissue occurs in over 95% of cases and live births have continued to climb exponentially, reaching well over 200 by now [2,4]. Shapira et al. gathered data from 3 clinical centers in Israel, Belgium and USA, reporting a live birth rate of 42% using the technique [5]. This was confirmed by very recent data from 5 leading European centers documenting the largest series so far, achieving an overall pregnancy rate of 38% and a live birth rate of 26% [6]. We report ovarian tissue transplantation using refrozen cortical strips from a cryopreserved whole ovary in a patient who was given high doses of pelvic radiotherapy for a stage IIIb rectal adenocarcinoma.

## 2. Detailed Patient Description

In October 2006, a 25-year-old woman presented with a stage IIIb rectal adenocarcinoma. Because of the advanced stage of the tumor, she required high doses of pelvic radiotherapy, with a cumulative radiation dose of 45 Gy combined with 5-fluorouracil chemotherapy to shrink the mass prior to surgery. Total mesorectal excision (TME) was then performed according to Heald’s surgical technique with lymph node dissection, and a J-shaped colic reservoir was created and anastomosed to the anal canal. Revision surgery was carried out a few days later for positive inferior margins and the patient needed a temporary protective ileostomy. Twelve cycles of FOLFOX 4 (5-fluorouracil, oxaliplatin and folinic acid) adjuvant chemotherapy were administered to complete her cancer treatment. In November 2007, liver metastasis was detected in segment VII and was surgically removed by segmentectomy. During this same operation, ileal continuity was reestablished. Genetic testing confirmed the patient was not a carrier of Lynch syndrome. All subsequent follow-up examinations were reassuring. 

Before undergoing any chemo- and radiotherapy, which are gonadotoxic, the patient was offered left whole ovary cryopreservation and her right ovary and tube were transposed outside the pelvic cavity to, respectively, preserve her fertility and protect it from radiation damage. The surgical and freezing procedures used to remove and cryopreserve the whole ovary were developed by our team and are described elsewhere [7,8]. The patient’s history is summarized in Table 1.

In March 2007, the menopausal status of the patient, aged only 26 years and experiencing hormone deprivation symptoms such as hot flushes and cessation of cycling, was confirmed by blood tests (estradiol < 10 ng/L; FSH > 100 IU/L), so hormone replacement therapy with 0.5 mg estradiol and 2.5 mg dydrogesterone was initiated. In 2019, the patient presented to our gynecology outpatients’ clinic seeking ovarian tissue transplantation. However, given the disseminated nature of her previous cancer and the estimated 3.4% incidence of ovarian involvement in colorectal cancer [9], preliminary tests were conducted to rule out any invasion of the cryopreserved organ by malignant cells. In order to conduct these tests, the whole ovary was thawed and cut into cortical strips (*n* = 53). At this stage, 4 cortical strips were used for minimal residual disease (MRD) screening by means of long-term xenografting, as well as histological and molecular analyses. Four more cortical strips were refrozen and subsequently rethawed to complete the MRD evaluation and for quality assessment of the tissue after refreezing. The remaining strips (*n* = 45) were refrozen while awaiting the results.

## 3. Results

The results of the MRD investigation and quality control of the tissue after refreezing were reassuring, so autologous transplantation of refrozen-rethawed cortical strips could be contemplated. However, since the uterus was located in the radiation field, endometrial receptivity had to be tested prior to reimplantation. The endometrium was able to thicken up to 9.6 mm, showing a triple-layer aspect upon estrogen therapy (4 mg estradiol valerate daily for 7 days, followed by 6 mg daily for 6 days). An endometrial biopsy was taken and anatomopathological analysis confirmed a well developed endometrium in the proliferative phase.

**Figure 1:**
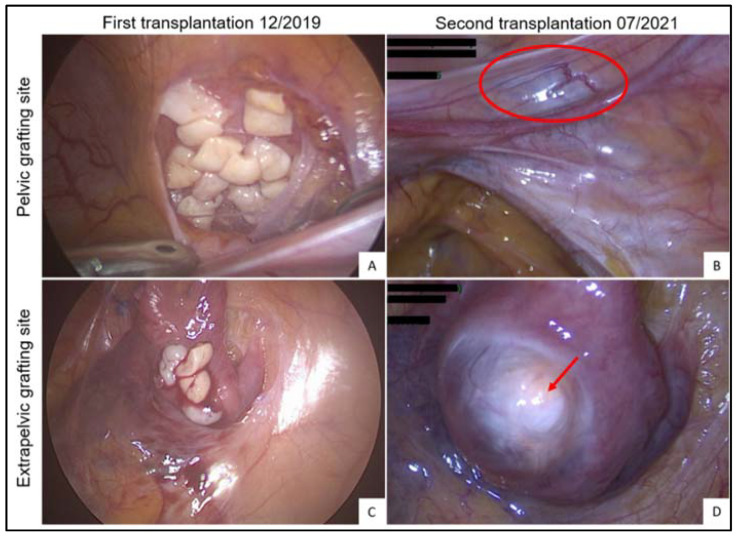
Ovarian tissue reimplantation. Pelvic grafting site of 18 refrozen-rethawed cortical strips inside a peritoneal window created on the right side of the bladder in December 2019 (**A**) and fibrotic aspect of the graft 1.5 years later in July 2021 (**B**) (red circle). Extrapelvic abdominal grafting site of 4 refrozen-rethawed cortical strips outside the irradiation field to the transposed tube in December 2019 (**C**) and functional aspect of the graft 1.5 years later with a visible antral follicle (**D**) (red arrow).

**Figure 2:**
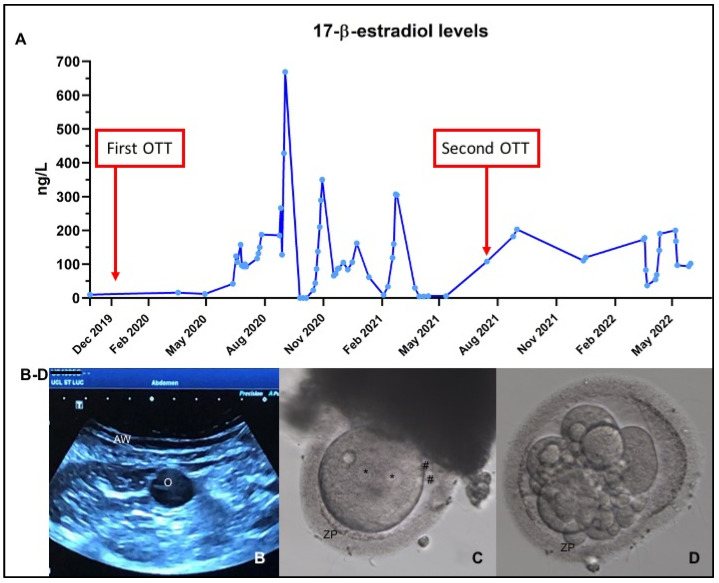
Ovarian function follow-up. (**A**) 17-β-estradiol levels. (**B**) Transabdominal ultrasound on the day of oocyte pick-up. (**C**) Fertilized oocyte on day 1, containing 2 pronuclei (*) and extruding 2 polar bodies (#). (**D**) Dividing embryo at the 6-cell stage on day 3 prior to transfer. AW: abdominal wall; O: oocyte; ZP: zona pellucida.

In December 2019, ovarian tissue was reimplanted by laparoscopy inside 2 peritoneal windows: (i) 18 strips were grafted to the pelvic cavity on the right of the bladder (Figure 1A); and (ii) 4 strips were grafted outside the irradiated pelvic cavity to the transposed tubal serosa (since the transposed ovary was atrophic and therefore too small to hold any ovarian fragments) (Figure 1B). The patient recovered menses 6 months later, as expected. Since natural conception was not possible in this case because no functional tubes remained, the patient was closely monitored by the IVF department. Ovarian function lasted 1.5 years, during which time 6 cycles were attempted (Table 2; Cycle 1–6). Controlled ovarian stimulation (COS) was applied in 4 of them, yielding 2 oocytes and one successful fertilization, which led to one fresh embryo transfer but no pregnancy (Figure 2B–D). By July 2021, the grafts were found to have stopped functioning for more than 3 consecutive months, with undetectable estradiol blood levels and cessation of cycling. It was therefore decided to retransplant all remaining refrozen cortical strips (*n* = 23). During this second laparoscopy, we were able to visualize a large antral follicle in the extrapelvic grafting site (Figure 1D). Ovarian activity was unexpected at the time of surgery and was confirmed by high estradiol levels reaching 108 ng/L (Figure 2A). Further proof of ovarian function from the first graft was the occurrence of menses 14 days after surgery. It is noteworthy that only fibrotic-looking tissue remained in the pelvic grafting site (Figure 1B), reflecting the absence of ovarian activity observed by transvaginal ultrasound in that location. Given these findings, all remaining cortical strips (*n* = 23) were reimplanted inside peritoneal windows in non-irradiated extrapelvic sites around the transposed right adnexa in July 2021. Since there was an overlap in ovarian function originating from the first graft at the time of the second transplantation, it was not possible to determine the time to ovarian recovery after the second transplant. Following this second reimplantation, 6 IVF cycles were attempted, 5 with COS (Table 2; Cycle 7–12). This resulted in the transabdominal retrieval of one fertilizable oocyte from the extrapelvic graft, but the embryo obtained was of poor quality and no transfer was performed. Ovarian function is still ongoing at the time of writing this paper.

## 4. Discussion

Whole ovary cryopreservation and transplantation has been proposed as an alternative to cortical strip freezing, since it could theoretically extend the longevity of ovarian grafts by transplanting the entire follicle pool and avoiding ischemic damage to the ovary thanks to vascular anastomosis [7,8,10,11]. Although several animal live births have been achieved in sheep with this procedure, its success is greatly dependent on microvascular reanastomosis, which requires high levels of surgical expertise, with the risk of losing the entire organ in the event of thrombosis [11,12,13,14]. Given the preclinical all-or-nothing nature of the procedure, we opted for reimplantation of cortical strips in the present study, since this has proved effective in the past and is no longer considered experimental [2,6,15]. Cortical strips also enable repeated transplantations, hence extending the fertile lifespan of the patient.

We hereby provide unprecedented proof that human ovarian tissue that has undergone whole ovary cryopreservation, following the protocol described by Martinez-Madrid et al. [7], is able to resume endocrine and exocrine ovarian function upon autologous ovarian tissue transplantation in the form of cortical strips. This confirms preclinical studies demonstrating the suitability of the latter cryopreservation protocol through histological, ultrastructural, and xenografting experiments upon thawing of frozen human whole ovaries [7,10,16].

Moreover, this is the first clinical proof of ovarian function reestablishment after reimplantation of refrozen-rethawed ovarian cortical strips issuing from a frozen-thawed whole ovary. Indeed, we demonstrate that ovarian tissue subjected to 2 freezing-thawing cycles is able to restore ovarian function upon autotransplantation. Recovery of ovarian function was assessed by various means, including recovery of menses, resumption of estradiol secretion, detection of follicle development by ultrasound and retrieval of 3 fertilizable oocytes after COS, which yielded 2 embryos. These findings confirm preliminary laboratory studies. Kristensen et al. reported the possibility of refreezing grafted ovarian tissue retrieved from a patient with a history of ovarian tissue cryopreservation and transplantation in the context of ovarian cancer [17]. The grafts were removed and refrozen once the patient had delivered healthy twin boys. Two pieces of refrozen ovarian grafted tissue were transplanted subcutaneously to an immunodeficient mouse. After 4 weeks, histological evaluation of the refrozen-grafted ovarian tissue revealed the presence of preantral-stage follicles in the recovered fragments [17]. Subsequently, our team was the first to conduct a pilot study using ovarian tissue from 4 patients [16]. Tissue from these patients was frozen in the form of a whole ovary with its vascular pedicle. When the whole ovaries were thawed, they were cut into cortical strips and divided into 4 experimental subgroups: (a) frozen-thawed non-grafted, (b) frozen-thawed xenografted, (c) refrozen-rethawed non-grafted, and (d) refrozen-rethawed xenografted. We found that mean follicle densities remained comparable between frozen-thawed and refrozen-rethawed non-grafted groups, as well as both grafted groups [16]. Furthermore, we did not detect any significant difference in atretic follicle rates between any of the 4 groups, and the ultrastructural quality of follicles appeared to be unaltered by the refreezing procedure [16]. Similar proportions of fibrosis were noted in frozen-thawed and refrozen-rethawed tissues and graft revascularization was unaffected by the number of freezing-thawing cycles [16]. Taken together, these results suggest that refrozen-rethawed ovarian tissue has the same functional characteristics as frozen-thawed ovarian tissue. In this paper, observations are based on the experience of one patient, but still prove the clinical feasibility of the approach. Nevertheless, its reproducibility requires further scrutiny.

The present study also highlights the deleterious impact of previous pelvic radiotherapy on ovarian tissue transplanted there. During the first ovarian transplantation attempt in December 2019, we reimplanted some fragments to the formerly irradiated pelvic cavity because the peritoneum appeared healthy and well vascularized. These ovarian fragments grafted to irradiated pelvic tissue were found to have been resorbed into fibrotic-looking tissue by the time of the second reimplantation 1.5 years later. No ovarian activity was detected by transvaginal ultrasound during this period, while follicle development was observed in the graft located outside the irradiation field, as visualized by transabdominal ultrasound. These findings shed light on a recent report in which the live birth rate declined to barely 8% in patients who had received targeted radiotherapy to the pelvis prior to transplantation [6]. Such bad outcomes may be related to poor neoangiogenic activity in ovarian grafts due to (i) a fibrotic reaction in irradiated pelvic tissue (including peritoneum), with poor residual vascularization, and (ii) irradiation of the uterus [6]. It is important to bear in mind that a cumulative radiation dose as low as 5 Gy has been found to increase the risks of miscarriage, low birth weight and premature birth [18]. Uterine reproductive capacity is more sensitive to radiation during childhood, making pregnancy inadvisable when uterine radiation reaches >25 Gy, while in adults irreversible injury may occur when the dose exceeds 45 Gy [18]. Nevertheless, another of our patients given high doses of pelvic radiotherapy with a cumulative radiation dose of 45 Gy for colorectal cancer, as in the present study, was able to deliver a healthy baby after ovarian tissue transplantation, despite giving birth prematurely at 32 weeks (data not shown). Overall, reimplanting ovarian tissue in patients who have undergone pelvic radiotherapy is possible, but the radiation dose and zone are key factors that must be considered prior to transplantation. Moreover, the grafting site should be away from the irradiated field, even if it appears healthy at first glance.

Our findings also show that ovarian tissue transplanted to peritoneum outside the pelvic cavity is able to resume ovarian function within a comparable time frame to grafting inside the pelvic cavity. This is in line with a recent study reporting similar outcomes in terms of numbers of mature oocytes aspirated, embryo development and live birth rates, whether the tissue was grafted to ovarian, pelvic or extrapelvic abdominal sites [19]. However, the authors stress that follicle maturation in extrapelvic locations needs to be triggered at a smaller-than-normal follicle diameter of approximately 13.5 mm [19].

Finally, we would like to emphasize that ovarian transposition should not be considered the only option for fertility preservation. Indeed, in the present study, the transposed ovary failed to maintain ovarian activity after being subjected to gonadotoxic regimens, including pelvic radiotherapy, and was completely atrophic at the time of the first ovarian tissue transplantation surgery 13 years later. Although ovarian transposition is able to maintain ovarian activity in some cases [20,21], its efficacy is unpredictable. In fact, the risk of developing premature ovarian insufficiency following ovarian transposition and pelvic radiotherapy is highly variable, ranging between 15% and 100% [20]. It is therefore strongly recommended that it be combined with other fertility preservation strategies [1,22]. Ovarian transposition is no longer recommended in women with a low ovarian reserve, at increased risk of ovarian metastasis, or in whom chemotherapy is planned as part of their treatment [22].

To conclude, the present study provides strong clinical evidence of the ability of ovarian tissue to withstand whole ovary cryopreservation and ovarian tissue refreezing, with resumption of menstrual cycling and conception of 2 embryos after the transplantation procedure. These findings are based on the experience of one patient, so further investigations are required to confirm reproducibility.

We also highlight the extremely poor outcomes of grafting ovarian tissue to previously irradiated peritoneum. Any history of pelvic radiotherapy should therefore be evaluated prior to transplantation and the grafting site designated away from the irradiated zone.

## Figures and Tables

**Table 1:** Outline of patient history.

Year	Patient History
**2006**	**October:** Diagnosed with colorectal adenocarcinoma ○ **5 October:** Whole ovary cryopreservation (left) and adnexa transposition (right) ○ **17 October–22 November:** Neoadjuvant pelvic radiotherapy (45 Gy) with chemotherapy (5-fluorouracil) **November:** Total mesorectum excision, with second revision surgery
**2007**	**February–September:** Adjuvant chemotherapy (12 cycles of FOLFOX 4) **March:** Iatrogenic menopause due to radio-chemotherapy **November:** Surgical removal of liver metastasis in S7
**2019**	**May:** Whole ovary thawing and dissection into cortical strips (*n* = 53) for MRD screening (*n* = 8) and refreezing of remaining cortical strips used for autotransplantation (*n* = 45) **December:** First transplantation of refrozen cortical strips to 2 distinct sites (*n* = 22): ○ Pelvic site: right paravesical peritoneal window (*n* = 18) ○ Extrapelvic: transposed right adnexa (*n* = 4)
**2020**	**June:** Recovery of endocrine ovarian function and menstruation **September:** Fresh embryo transfer, but no pregnancy
**2021**	**July:** Second transplantation of refrozen cortical strips to 2 peritoneal windows around the transposed right adnexa (*n* = 23)
**2022**	Graft function ongoing

Gy: Gray; FOLFOX 4: 5-fluorouracil, oxaliplatin and folinic acid; S7: liver segment VII; MRD: minimal residual disease.

**Table 2:** In vitro fertilization history.

Cycle Number	Date	COS Protocol (Total Dose)	Hormone Level at Ovulation Trigger, When Appropriate		Follicle(s) Visualized at US? (*n*)	Response to Stimulation?	OPU	Embryo	Transfer
			E2 (ng/L)	LH (IU/L)					
**5 December 2019: First OTT**									
1	27 June 2020	FSH (1050 IU); GnRH antagonist (2.25 mg)	93	15	Yes (1)	No	-	-	-
2	26 August 2020	FSH (1050 IU); GnRH antagonist (2.25 mg)	669	20.3	Yes (2)	Yes	1 oocyte and 1 EF (TA)	1	1 (fresh)
3	23 October 2020	FSH (1600 IU); GnRH antagonist (1.75 mg)	350	17.1	Yes (2)	Yes	1 oocyte (TA)	0	0
4	17 November 2020	No stimulation	105	47	No	-	-	-	-
5	25 January 2021	FSH (1800 IU); GnRH antagonist (1.5 mg)	305	23.3	Yes (2)	Yes	1 EF * (TV) and 1 extrapelvic US image that disappeared	-	-
6	24 March 2021	No stimulation	6	76	No	-	-	-	-
**15 July 2021: Second OTT**									
7	15 September 2021	FSH (1500 IU); GnRH antagonist (1.25 mg)	94	14	Yes (1)	Yes	1 EF (TA)	-	-
8	20 October 2021	FSH (600 IU); GnRH antagonist (0.25 mg)	91	12.2	Yes (1)	Yes	Patient requested stopping the cycle	-	-
9	29 November 2021	No stimulation	120	51	No	-	-	-	-
10	19 March 2022	FSH (1200 IU); GnRH antagonist (0.75 mg)	37	9	Yes (1)	No	-	-	-
11	11 April 2022	Ovulation trigger only	141	32	Yes (1)	-	1 EF (LPS; extrapelvic site)	-	-
12	7 May 2022	FSH (600 IU); GnRH antagonist (0.5 mg)	97	10	Yes (1)	Yes	1 oocyte (TA)	1	0

COS: controlled ovarian stimulation; E2: 17-β-estradiol; LH: luteinizing hormone; US: ultrasound; OPU: oocyte pick-up; OTT: ovarian tissue transplantation; FSH: follicle-stimulating hormone; GnRH: gonadotropin-releasing hormone; IU: international unit; EF: empty follicle; TV: transvaginal; TA: transabdominal; LPS: laparoscopic puncture. *: TV puncture of a persistent US image most likely corresponding to a peritoneal cyst rather than an ovarian follicle.

## Data Availability

Data on any of the subjects in this study have not been previously published unless specified. Data will be made available to the editors of the journal for review or query upon request.
